# Comparison of Neurocognitive Functioning and Fine Motor Skills in Pediatric Cancer Survivors and Healthy Children

**DOI:** 10.3390/cancers14235982

**Published:** 2022-12-03

**Authors:** Nadezda Chipeeva, Alena Deviaterikova, Elena Glebova, Elizaveta Romanova, Alexander Karelin, Vladimir Kasatkin

**Affiliations:** 1 Research Institute for Brain Development and Peak Performance, Peoples Friendship University of Russia, 117198 Moscow, Russia; 2 Dmitry Rogachev National Medical Research Center of Pediatric Hematology, Oncology, and Immunology, 117198 Moscow, Russia

**Keywords:** pediatric brain tumor, tumors of hematopoietic and lymphoid tissues, neurocognitive functioning, fine motor skill

## Abstract

**Simple Summary:**

Various impairments in neurocognitive and fine motor skills represent a common side effect of both pediatric tumors and their therapy in children. Here we compare the neurocognitive and fine motor skills deficits manifested in the group with pediatric tumors against the background of the healthy control group. Worse impairments of neurocognitive and fine motor skill were revealed in pediatric brain tumor survivors as compared with hematopoietic and lymphoid tissue tumor survivors. Significant differences between the cognitive functions of females and males were detected only in the pediatric brain tumor survivors group.

**Abstract:**

Background: The late treatment outcomes of pediatric brain tumors and of hematopoietic and lymphoid tissue tumors are an important focus of both rehabilitation and research. Neurocognitive and motor disorders induce further learning problems impeding social-emotional adaptation throughout a whole lifespan. Core deficits in short-term and working memory, visuospatial constructional ability, verbal fluency, and fine motor skills underlie distorted intellectual and academic achievement. This study aimed to assess the individual differences in cognitive ability and fine motor skills of pediatric tumor survivors and the age-matched healthy controls. Methods: A total of 504 tumor survivors after treatment and 646 age-matched healthy controls underwent neurocognitive and fine motor assessments. Findings: The group of tumor survivors scored significantly worse in both neurocognitive and fine motor skill in compared with the healthy control group. The pediatric brain tumor survivors (PBT group) performed worse in cognitive (*p* < 0.001 for verbal fluency and *p* < 0.001 for visuospatial constructional ability) and motor tests (*p* < 0.001) compared to the healthy controls. Hematopoietic and Lymphoid Tissues tumors survivors (THL group) performed worse in verbal fluency (*p* < 0.01) and visuospatial constructional test (*p* < 0.001) compared to the control group. Furthermore, the PBT group had worse results in visuospatial constructional ability (*p* < 0.05) and fine motor (*p* < 0.001) ability than the THL group. Significant differences between females and males were found in fine motor test performance in the PBT group (*p* < 0.05), as well as in verbal fluency (*p* < 0.01) and visuospatial constructional ability (*p* < 0.01) in the control group. Neurocognitive and fine motor skill characteristics in the THL group did not correlate with age.

## 1. Introduction

Rapid progress in modern medicine has led to a steady increase in cancer survival rates. The number of people who have successfully completed treatment and can return to normal life is increasing every year. At present, the life expectancy of children who have survived cancer is assumed to differ only slightly from that of their healthy classmates [1]. Despite remarkable prognoses for children’s survival and their increased life expectancy, modern treatment remains highly toxic and long, which almost always leads to serious consequences that may manifest themselves even several years after treatment has already been completed—the so-called late effects [2]. Late effects of treatment can be expressed both in somatic symptoms, malfunction of the organs and their systems, as well as in various disorders of cognitive and motor functions [3]. Research shows that the earlier the onset of the disease is, the higher the risk of cognitive and motor deficits [4,5,6,7]. These deficits can negatively affect schooling and the subsequent quality of life of those who have experienced cancer in childhood [8,9,10]. Childhood cancer survivors are a special risk group for impaired psychosocial skill, marital and employment status [11]. Only 66% of all childhood cancer survivors are employed. A recent meta-analysis shows the lowest rate of employment for central nervous system tumor survivors, patients treated with cranial-radiotherapy and hematopoietic stem-cell transplantation (with employment rates of 51%, 53% and 56%, respectively) [12]. Thereby, late sequelae cognitive and motor impairments secondary to their primary disease, the time of its onset, treatment and adjuvant therapy significantly affect the whole life of cancer survivors.

Pediatric brain tumors and childhood tumors of hematopoietic and lymphoid tissues account for the lion’s share of all pediatric cancer (leukemias, at 34%, and lymphomas, at 12%) [13]. A lot of research has demonstrated negative outcomes for pediatric brain cancer survivors, among which a decrease in cognitive, visual-motor, and visuospatial functioning were the most common [14]. This might be related to several factors: location of tumor as well as tumor origin and progression, type of therapy (for instance resection of a part of the brain for the pediatric cancer of the central nervous system), chemotherapy and radiation therapy, the consequences of treatment and hospitalization, age and time of the diagnosis, duration of treatment, and many other factors [15,16,17]. A systematic review showed that tumor histopathology and subsequent adjuvant therapy as well as the age of diagnosis influenced later neuropsychological difficulties in CNS tumor survivors [18]. In particular, medulloblastoma survivors showed worse intelligence performance, and lower levels of attention, memory and executive function than astrocytoma survivors [18,19]. The study found that survivors of CNS tumors generally had lower cognitive ability scores compared to the general population [18,20]. A meta-analysis of the long-term neurocognitive outcomes of pediatric posterior fossa brain tumor patients revealed that their full-scale intelligence, attention and concentration, motor abilities, visual–spatial skills, visual and verbal memory, information processing speed, and academic achievement were significantly poorer than those in the healthy control. Moreover, the essential factors influencing the severity of neurocognitive impairment were the young age of disease onset and adjuvant radiation therapy [21,22,23].

The decrease in neurocognitive abilities is most naturally associated with the brain structures damaged during cancer therapy. In particular, the structures of the cerebellum and hippocampus yield the most significant impact on motor and cognitive abilities. Neuroimaging studies showed that the impaired cerebellum neuron network can lead to cerebellar cognitive affective syndrome (CCAS) [24,25]. This syndrome manifests itself through deficits in executive functions (verbal fluency, planning, working memory), impaired spatial cognition (visuospatial perception and memory), linguistic ability and disorders (dysprosody, agrammatism, and mild anomia), as well as through personality and affective disorders [26,27]. Since hippocampus is involved in memory processes and memorization, hippocampal impairment is related to memory decline [28,29].

Acute lymphoblastic leukemia (ALL), as well as other types of childhood intracranial brain oncology, can result in decreased cognitive functions in children after treatment [30]. A meta-analysis of neurocognitive functioning in lymphoblastic leukemia survivors showed that patients with ALL scored significantly worse in full-scale IQ and verbal IQ tests. Moreover, lymphoblastic leukemia survivors’ working memory (Digit Span Forward and Backward), information processing speed, verbal and visual memory, fine motor functioning, and executive function were lower than those in the healthy control group. The results described in this meta-analysis demonstrate the long-term effects of cognitive impairment for ALL survivors after chemotherapy without cranial radiation [31]. This therapy protocol is considered relatively spared for cognitive functioning compared with protocols including cranial radiation [32,33,34,35]. Even though the treatment protocols involving specific medications may be less or even non-toxic, they can still bring about severe cognitive decline [32,33]. For instance, methotrexate treatment can have detrimental effects on neurocognitive functioning [34]. In addition, this treatment negatively affects the conductivity of the white matter of the brain [35,36].

Most studies of neurocognitive and motor functioning were conducted on small or moderate samples [37,38]. The present study aims to assess the differences in cognitive functioning between Pediatric Brain Tumors and Hematopoietic and Lymphoid Tissues Tumor survivors, and the age-matched healthy control group. Young age at diagnosis is an essential factor that affects the neurocognitive outcomes. At the same time, the effects of gender and sex differences are unclear. Some works have reported worse results for female tumor survivors than for male ones, while other studies do not support these findings [39]. Moreover, some research lacked a comparison with the healthy control group.

This work aims to fill in the gap in systematic understanding of the detrimental effects of pediatric cancer and its treatment on a large and varied sample in terms of cancer types studied at the background of the no lesser representative sample of healthy controls applying a unified metrics and assessing the correlations with age and gender.

The data obtained in the present study will be useful for updating the existing data, since earlier studies did not investigate large clinical samples. We hypothesized that the group with Pediatric Brain Tumor would demonstrate worse cognitive performance than the group with Hematopoietic and Lymphoid Tissue Tumors, and that both groups would perform worse than healthy controls. In addition, possible differences in neurocognitive outcomes between females and males in each group were studied.

## 2. Materials and Methods

### 2.1. Participants

The experimental group included pediatric tumor survivors aged from 6 to 17 who were recruited from the Russkoe Pole Clinical Research Rehabilitation Center of Dmitry Rogachev National Medical Research Center of Pediatric Hematology, Oncology, and Immunology. The control group included healthy children of the same age recruited from Moscow and the Moscow region. All participants spoke Russian as their mother tongue. The experimental sample included groups of children who successfully completed treatment from Pediatric Brain Tumors (PBT), tumors of hematopoietic and lymphoid tissues (THL) and healthy controls (Control).

All patients received appropriate treatment. Pediatric brain tumors survivors (PBT) received treatment according to the protocol of the HIT MED group. The THL group included patients with the following diagnoses: non-Hodgkin lymphoma, acute myeloblastic leukemia, Hodgkin lymphoma, Langerhans’ cell histiocytosis and acute lymphoblastic leukemia. Non-Hodgkin lymphoma and acute myeloblastic leukemia (AML) survivors were treated according to the protocols of the BFM group. The treatment of Hodgkin lymphoma survivors followed the protocols of the GPOH group. Langerhans’ cell histiocytosis survivors received treatment according to the LCH protocol. Acute lymphoblastic leukemia survivors’ treatment was based on the protocols of the BFM and Moscow–Berlin groups.

The legal representatives of children younger than fifteen gave written informed consent before the initial tests. The experiment was approved by the Ethics Committee of Dmitry Rogachev National Medical Research Center of Pediatric Hematology, Oncology and Immunology (protocol number 10/2017 of 12.12.2017) and was run according to Helsinki Declaration.

### 2.2. Measures

Neuropsychological measures used in the study were reliable, valid, and had age-adjusted normative scores.

To assess short-term and working memory, the Wechsler Memory Scale, Third Edition Digit Span test was used [40]. In brief, the test consisted of two subtests, namely forward and backward spans. Each participant listened to several digits (one digit per second) and repeated it forward or backward. The first span included two digits, the last one included nine digits for the forward span, and the last one included eight digits for the backward span. Each task also contained 2 samples. For each sample, 1 point was given if everything was correct and 0 points if the answer was wrong or there was no answer. For each task, the score was summed up for 2 samples. The child was presented with both samples, even if the child has successfully completed the first sample. After two wrong answers, the second part of the test was also interrupted. The total score was calculated by summing the points for all the samples of each subtest. The maximum total score of each subtest was 16 points (Digit Span Forward and Digit Span Backward, respectively).

To the assess the visuospatial constructional ability, the Rey–Osterrieth Complex Figure (ROCF) was used [41,42]. The participants had to reproduce a complex geometric figure from the sample of the Ray–Osterrieth figure for copying. The psychologist marked the time as soon as the subject began to copy the figure. If the participant had been copying the figure for more than 10 min, the psychologist interrupted the test. The main criterion for scoring was the accuracy of the copied part of the figure and its exact location relative to the overall picture. The maximum score for each element of the figure was 2 points, the minimum is 0 points. The maximum number of points that a participant could receive for a completed task was 36 points.

Controlled Oral Word Association Task (COWAT) was used to evaluate verbal fluency [43]. The main purpose was to evaluate spontaneous production of words within a limited amount of time (one minute for each letter). We suggested three letters for the participants, these were three Russian letters—‘∏’, ‘O’, ‘C’ (English letters—‘p’, ‘o’, ‘s’). To assess the participants’ performance on verbal fluency, the psychologist calculated the total number of words produced across all three trials for each letter, minus any unacceptable responses. Unacceptable responses occurred when a patient repeated the previous response or made an error by including a word that starts with the wrong letter, or other rule violation. The final scores were the total numbers of words beginning with each letter.

The Grooved Pegboard test was used to assess fine motor skills, eye–hand coordination, and motor speed [44]. The apparatus for this test consisted of a metal panel with a 5 by 5 matrix of keyhole-shaped holes in various orientations. The participants were instructed to insert pegs using only one leading hand in a prescribed order, going as quickly as possible. The raw score of the test was calculated as the amount of time in seconds that it took the participant to place the pegs into all the holes on the board. Raw scores were used in the current study.

### 2.3. Statistical Analyses

The Shapiro–Wilk test was used to assess the normality of data distribution. All neurocognitive tests scores were not normally distributed. ANOVAs/Kruskal–Wallis were used to compare the differences in neurocognitive scores between two clinic groups and the control group; individual comparisons were assessed using post hoc Dunn test with p-adjusted Bonferroni correction.

Wilcoxon rank sum test with p-adjusted Bonferroni correction for independent samples was used to compare the differences in neurocognitive functioning between females and males in two clinic groups and the control group.

Spearman’s correlation analysis was applied to reveal the relationship between age and neurocognitive and fine motor measures. Correction for multiple comparisons was made using the FDR method [45].

Statistical analysis was performed using the Statistical Programming Environment R with packages “psych”, “rcompanion”, “rstatix”, “ggplot”, “corrplot”and JASP.

## 3. Results

The sample included 1150 participants: 137 PBT survivors, 367 THL survivors and 646 age-matched healthy controls. Table 1 provides the descriptive statistics of the participants’ demographic characteristics and neurocognitive measures by the clinic groups and the control group.

### 3.1. Comparison of Neurocognitive Outcomes by Clinic Group

Mean score and standard deviation of neurocognitive test scores and comparisons between the participants’ groups are presented in Table 2. Overall, the PBT and THL survivors did worse in the neurocognitive tests worse than the healthy control group. Significant differences were found in the Digit Span Forward (*p* = 0.0006, η^2^ = 0.0111), COWAT (*p* = 0.000004, η^2^ = 0.0199), the Rey–Osterrieth Complex Figure (*p* = 1.59 × 10^−12^, η^2^ = 0.0456) and Grooved Pegboard tests (*p* = 8.36 × 10^−20^, η^2^ = 0.0749).

**Figure 1:**
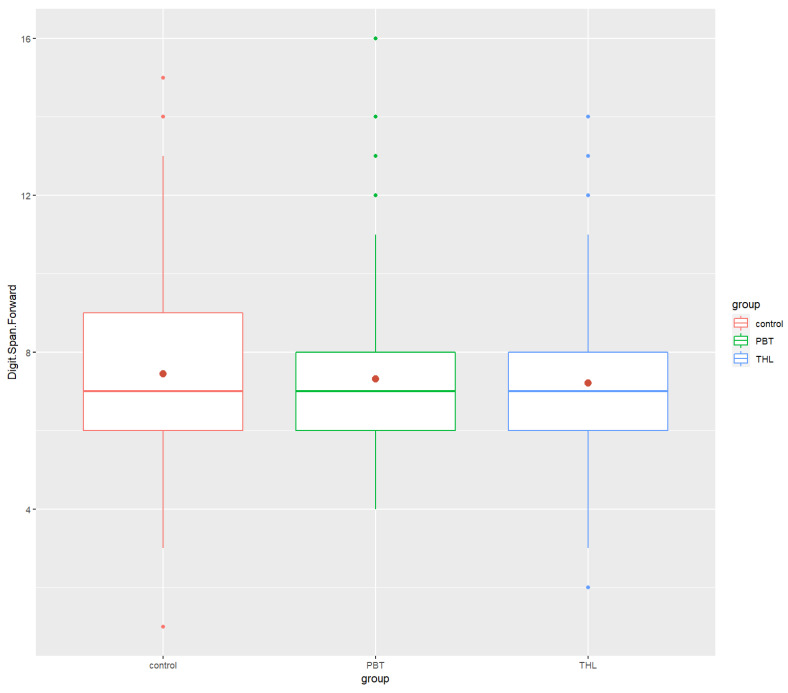
Boxplots of Digit Span Forward for the healthy control group and the diagnosis groups. Control—healthy control group, PBT—pediatric brain tumor survivors, THL—hematopoietic and lymphoid tissues tumor survivors. Red dot—mean test score for each group.

**Figure 2:**
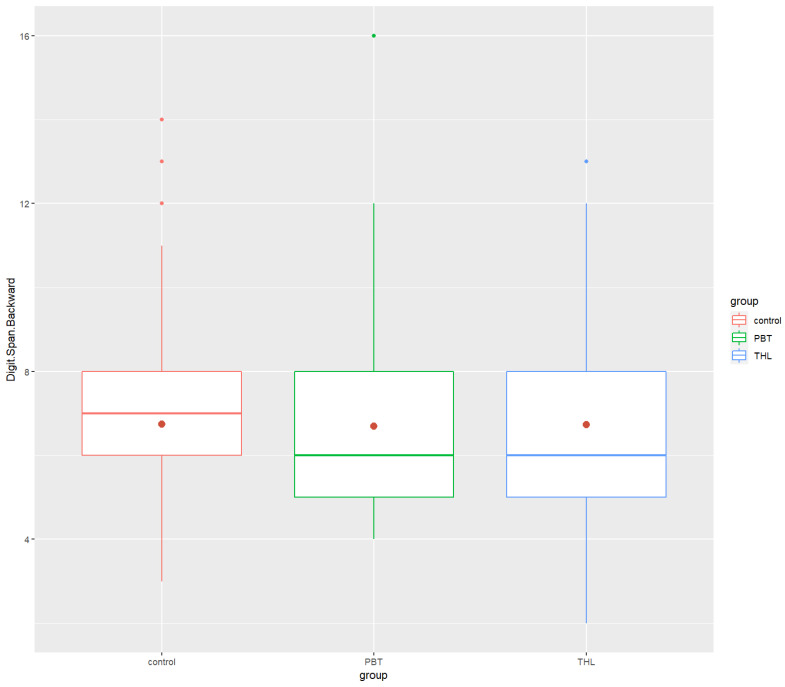
Boxplots of Digit Span Backward for the healthy control group and the diagnosis groups. Control—healthy control group, PBT—pediatric brain tumor survivors, THL—hematopoietic and lymphoid tissues tumor survivors. Red dot—mean test score for each group.

**Figure 3:**
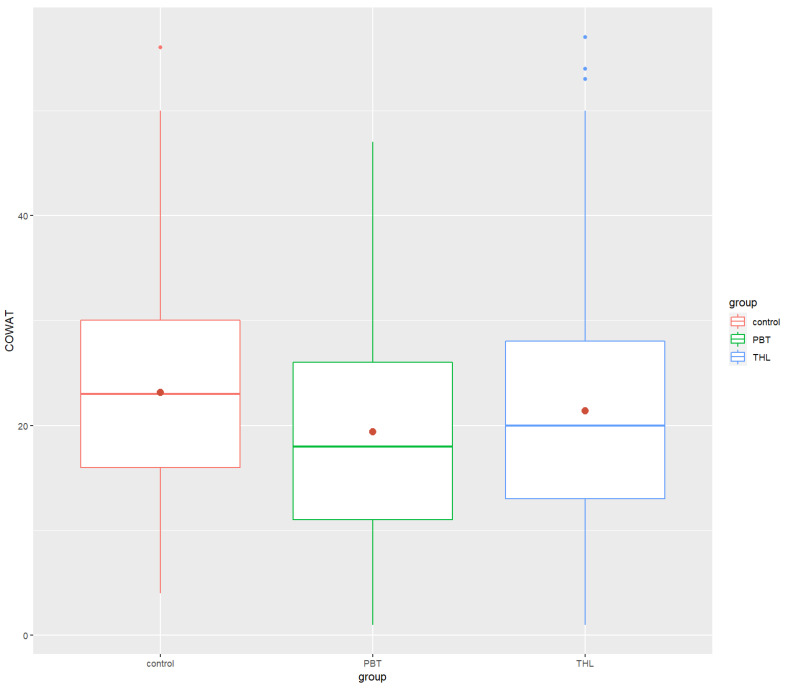
Boxplots of COWAT for the healthy control group and the diagnosis groups. Control—healthy control group, PBT—pediatric brain tumor survivors, THL—hematopoietic and lymphoid tissues tumor survivors. COWAT—Controlled Oral Word Association Task. Red dot—mean test score for each group.

**Figure 4:**
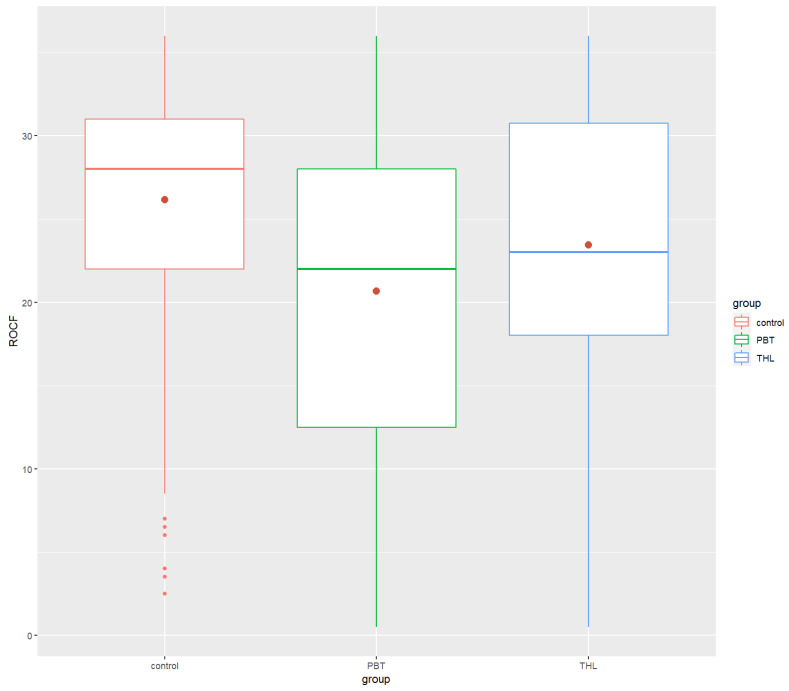
Boxplots of ROCF for the healthy control group and the diagnosis groups. Control—healthy control group, PBT—pediatric brain tumor survivors, THL—hematopoietic and lymphoid tissues tumor survivors. COWAT—Controlled Oral Word Association Task. ROCF—the Rey–Osterrieth Complex Figure. Red dot—mean test score for each group.

**Figure 5:**
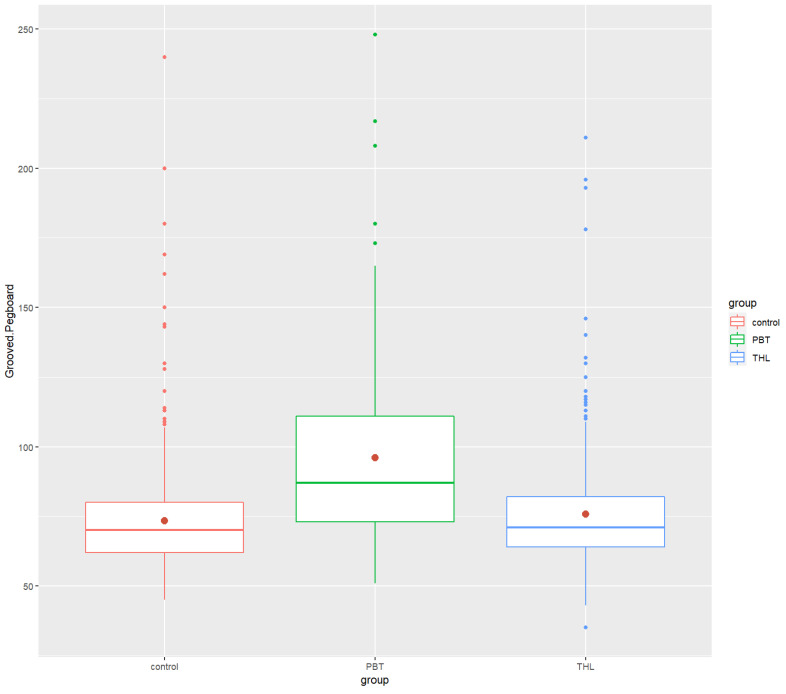
Boxplots of Grooved Pegboard for the healthy control group and the diagnosis groups. Control—healthy control group, PBT—pediatric brain tumors survivors, THL—tumors of hematopoietic and lymphoid tissues survivors. COWAT—Controlled Oral Word Association Task. Red dot—mean test score for each group.

Post hoc analyses revealed that the PBT group’s performance in neurocognitive tests was significantly lower than that of the control group in the COWAT (*p*-value = 0.0000534 ****), ROCF (*p* = 5.65 × 10^−10^ ****) and Grooved Pegboard (*p* = 3.08 × 10^−20^ ****). There were significant differences between the THL and the control groups in Digit Span Forward (*p* = 0.000474 ***), COWAT (*p* = 0.00128 **) and ROCF (*p* = 6.24 × 10^−7^ ****). The THL survivors did worse in the tests than the control group. Finally, significant differences between the PBT and THL groups were found in only ROCF (*p*-value = 2.84 × 10^−2^ *) and Grooved Pegboard (*p* = 2.18 × 10^−14^ ****), exclusively. The PBT group performed task in ROCF test worse and spent more time doing the Grooved Pegboard test than the THL group. All the findings were in the small effect size range, excluding the Grooved Pegboard results, which had a moderate effect size. Figure 1, Figure 2, Figure 3, Figure 4 and Figure 5 show the boxplots for the neurocognitive tests.

### 3.2. Comparison of Neurocognitive Measures by Clinic Group between Females and Males, and the Relationship with Age

In the healthy control group, the females performed better than the males in the COWAT (M/females = 24.3 and M/males = 21.9, *p* = 0.003 **, respectively) and ROCF tests (M/females = 27.0 and M/males = 25.3, *p* = 0.005 **, respectively). Among the clinic groups, significant differences were found for females (M/females = 91.5) and males (M/males = 101) from the PBT survivors group in the Grooved Pegboard test (*p* = 0.043 *). Significant differences between female and male survivors in the THL group were not found (Table 3).

**Figure 6:**
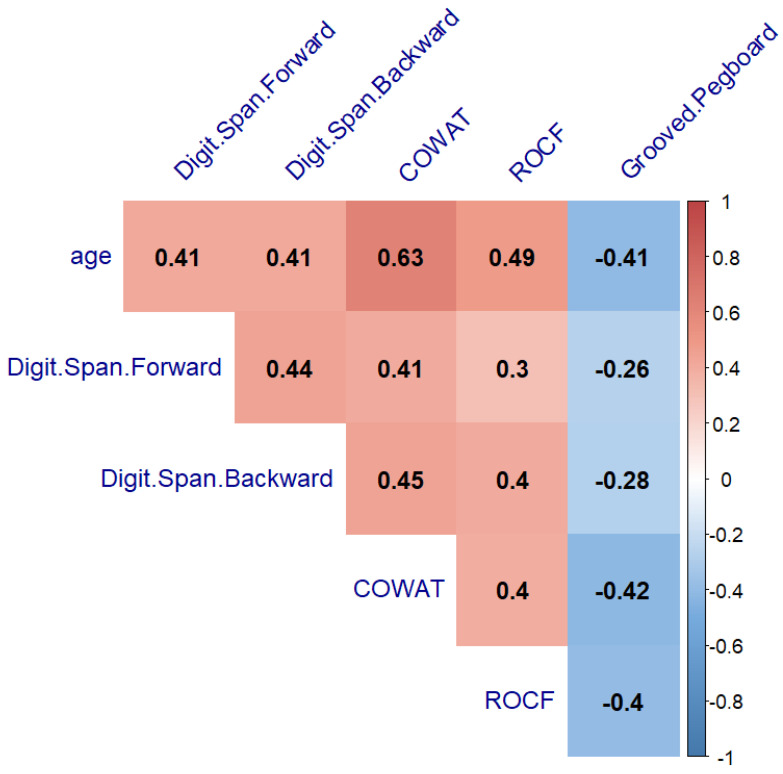
Heatmap of neurocognitive test results and age relationship in the healthy control group showing Spearman’s correlation after the FDR correction. COWAT—Controlled Oral Word Association Task, ROCF—the Rey–Osterrieth Complex Figure.

**Figure 7:**
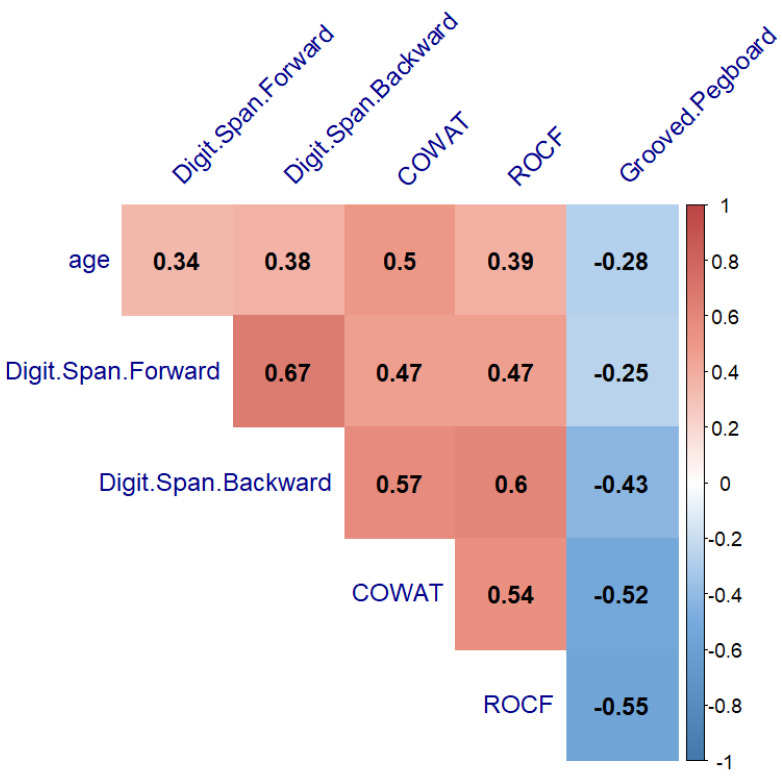
Heatmap of neurocognitive test results and age relationship in Pediatric Brain tumor group showing Spearman’s correlation after FDR correction. COWAT—Controlled Oral Word Association Task, ROCF—the Rey–Osterrieth Complex Figure.

**Figure 8:**
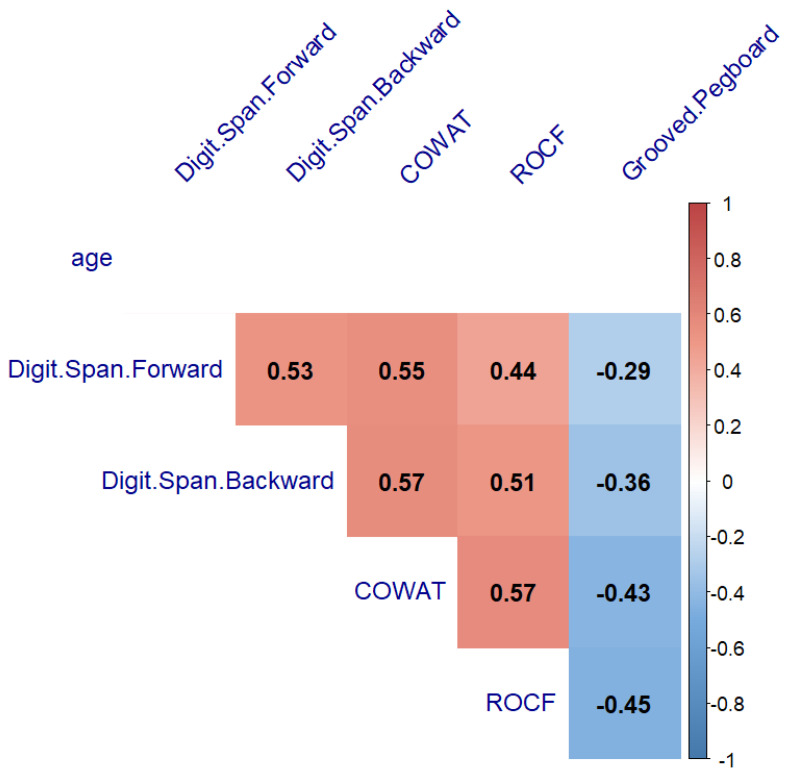
Heatmap of neurocognitive test results and age relationship in hematopoietic and lymphoid tissues tumor group showing Spearman’s correlation after FDR correction. COWAT—Controlled Oral Word Association Task, ROCF—the Rey–Osterrieth Complex Figure; THL—hematopoietic and lymphoid tissues tumor group.

The next step to verify our hypothesis was to search for the relationship between age with neurocognitive performance and fine motor ability. A significant correlation between age and neurocognitive performance was found in the healthy controls and PBT survivors (from r = −0.41 to r = 0.63, *p* < 0.05, and from r = −0.28 to r = 0.5, *p* < 0.05) (Figure 6 and Figure 7, respectively). The THL group’s neurocognitive performance did not correlate with age (*p* > 0.05) (Figure 8).

## 4. Discussion

Aiming to fill the lack of a large-cohort systematic study of cancer survivors against the background of healthy controls, here we reveal poorer neurocognitive characteristics and fine motor skills in childhood cancer survivors studied on the basis of a large and varied sample as compared to a normal sample of the same age (6–17 years). Furthermore, significant differences were found in the Digit Span Forward, Controlled Oral Word Association Task, the Rey–Osterrieth Complex Figure and Grooved Pegboard tests. The obtained results confirm data obtained previously showing a decline in the executive functions of the short-term memory, working memory and verbal fluency, as well as visual–spatial perception, and fine motor skills in survivors of pediatric tumors [46,47,48,49].

Impaired fine motor skills and cognitive functioning in lymphoblastic tumors survivors are related to chemotherapy treatment. Several survivors who underwent chemotherapy did not have motor impairments and preserved the integrity of both corticospinal tracts unlike other patients after a course of chemotherapy [50]. This could be associated with genome sensitivity and persistent neuropathy in the survivors [51]. Fine motor skills, intellectual functioning, attention, executive functions, and verbal learning often deteriorated after childhood posterior fossa tumor [52]. Despite a high survival rate with this type of cancer, the survivors face posterior fossa syndrome and problems with balance and movement [53], as well as the deficit in executive functions [20]. According to the pertinent literature, there is a network connecting cerebellum areas and frontal with prefrontal areas [19,54,55].

Our findings confirm the initial hypothesis that the neurocognitive test results of posterior fossa tumors survivors would be worse than those of lymphoblastic survivors. Significant differences were found in the tests of visuospatial function (the Rey–Osterrieth Complex Figure) and fine motor skills (Grooved Pegboard). Thus, neurocognitive functioning is sensitive to the type of tumor, tumor grade and treatment, and adjuvant therapy. Malignant tumors require intracranial irradiation in combined with adjuvant chemotherapy [56,57]. Treating of Posterior fossa tumors often involves the increased irradiation of this region and resection of the affected area when possible [18,58].

The present study demonstrated significant differences in neurocognitive performance between girls and boys only in the pediatric brain cancer survivors group yielding negligible differences in other tumor types. The females had better scores in the fine motor skills test. These data contradict the idea that female gender may be a risk factor for neurocognitive outcomes, yet confirm other risk factors [39,59,60]. Better results may be connected with the advanced cognitive development of females as compared with males in early childhood and adolescence [61]. In addition, tumor size and treatment type were demonstrated to be more critical for neurocognitive outcomes than gender [60].

The age factor was revealed to be particularly significant for the childhood cancer survivors group. No significant relationship with age was found in the group with lymphoblastosis. Pediatric tumors can delay cognitive and motor development in the sensitive period of these functions in the childhood [19]. However, the lack of influence of the age factor on the cognitive functions and fine motor skills of the lymphoblastic survivors group requires further research.

Our study, conducted on a fairly large sample of participants, confirms the results of other studies based on smaller samples showing that children who have survived cancer have cognitive and motor deficits [14,16]. Meanwhile, the large and varied clinical sample both in terms of the cancer survivor group and the control group could provide the solid rationale for improving and updating rehabilitation procedures aiming at restoring the neurocognitive and fine motor skills and potentially choosing the best pipeline for each individual patient, thus leading to potential improvements in cognitive outcomes and quality of life.

## 5. Limitations

The present study has several limitations. First, the incomplete information on the treatment protocols for cancer survivors groups limits the study’s findings. Another limitation is the lack of data on the socioeconomic status of the participants, which could have contributed to the research conclusions. The third limitation is related to the difficulty in the process of collecting some neurocognitive test results, such as the Rey–Osterrieth Complex Figure, COWAT, and Grooved Pegboard tests, as the results, to a large extent, depend on the qualification of the psychologist who conducts them.

## 6. Conclusions

This paper aimed to systematically review the evidence of cognitive and motor deficits of varying severity in children who survived cancer. Our results show that the motor deficits are more pronounced among the pediatric brain tumor survivors than among the children who have survived oncological diseases of the blood system. There is cognitive decline in both groups, but these changes manifest themselves with different degrees of severity. These cognitive impairments can have a significant impact on the school success of children who have survived cancer and returned to school, so these children need additional rehabilitation guidance aimed at improving cognitive and motor functions after the treatment itself is completed. In conclusion, the present study updates the current data on the neurocognitive development among groups of survivors with pediatric brain cancer and tumors of hematopoietic and lymphoid tissues. One of the advantages of the study is the large group of cancer survivor participants studied at the background of the healthy control group of no lesser size.

## Figures and Tables

**Table 1:** Descriptive statistics of demographic characteristics and neurocognitive test scores of healthy controls (*n* = 646), pediatric brain tumor survivors (*n* = 137), and hematopoietic and lymphoid tissues tumor survivors (*n* = 367).

Demographic Characteristics and Methods	Descriptive Statistics	Control	PBT	THL
Age				
	Mean (SD)	11.36 (2.79)	11.39 (2.97)	10.56 (3.08)
	Range	7–16	6–17	6–17
Sex				
	Females	310	72	168
	Males	336	65	199
Digit Span Forward				
	Mean (SD)	8.14 (1.80)	7.94 (1.87)	7.70 (1.87)
	Median	8	8	8
	Min/Max	1.0/15	4.0/14	3.0/14
Digit Span Backward				
	Mean (SD)	6.75 (1.82)	6.70 (2.12)	6.73 (1.88)
	Median	7.0	6	6
	Min/Max	3.0/14	4.0/16	2.0/13
COWAT				
	Mean (SD)	23.17 (9.39)	19.39 (10.39)	21.37 (11.13)
	Median	23.0	18	20
	Min/Max	4.0/56	1.0/47	1.0/57
ROCF				
	Mean (SD)	26.15 (6.60)	20.66 (9.40)	23.44 (7.97)
	Median	28.0	22	23
	Min/Max	2.5/36	0.5/36	0.5/36
Grooved Pegboard				
	Mean (SD)	73.39 (18.65)	96.01 (32.87)	75.79 (20.62)
	Median	70.0	87	71
	Min/Max	45.0/240	51.0/248	35.0/211

Control—healthy controls, PBT—pediatric brain tumors survivors, THL—hematopoietic and lymphoid tissues tumor survivors. COWAT—Controlled Oral Word Association Task, ROCF—the Rey–Osterieth Complex Figure.

**Table 2:** Mean group comparisons of neurocognitive test scores among the healthy controls (*n* = 646), pediatric brain tumor (*n* = 137), and hematopoietic and lymphoid tissues tumor survivors (*n* = 367).

Neurocognitive Measures	Control		PBT		THL		Kruskal–Wallis One-Way ANOVA		Dunn Test		
	Mean (SD)	Median	Mean (SD)	Median	Mean (SD)	Median	*p*-Value	η^2^	Control—PBT	Control—THL	PBT—THL
Digit Span Forward	8.14 (1.80)	8	7.94 (1.87)	8	7.70 (1.87)	8	0.0006 ***	0.0111	0.349 (ns)	0.000474 ***	0.965 (ns)
Digit Span Backward	6.75 (1.82)	7	6.70 (2.12)	6	6.73 (1.88)	6	0.653 (ns)	−0.00100	0.374 (ns)	0.642 (ns)	0.596 (ns)
COWAT	23.17 (9.39)	23	19.39 (10.39)	18	21.37 (11.13)	20	0.000004 ***	0.0199	0.0000534 ****	0.00128 **	0.250 (ns)
ROCF	26.15 (6.60)	28	20.66 (9.40)	22	23.44 (7.97)	23	1.59 × 10^−12^ ***	0.0456	5.65 × 10 ^−10^ ****	6.24 × 10^−7^ ****	2.84 × 10^−2^ *
Grooved Pegboard	73.39 (18.65)	70	96.01 (32.87)	87	75.79 (20.62)	71	8.36 × 10^−20^ ***	0.0749	3.08 × 10 ^−20^ ****	3.90 × 10^−1^ (ns)	2.18 × 10^−14^ ****

Medium-to-large effect size (η^2^ ≥ 0.06). Dunn’s test as a post-hoc test for significant Kruskal–Wallis test and adjusted *p*-value with Bonferroni correction are shown. Control—healthy controls, PBT—pediatric brain tumors survivors, THL—hematopoietic and lymphoid tissues tumor survivors. COWAT—Controlled Oral Word Association Task, ROCF—the Rey–Osterrieth Complex Figure. ****/***—Statistically significant difference, *p* < 0.001, ** *p* < 0.01, * *p* < 0.05, ns—not significant.

**Table 3:** Mean group comparisons of neurocognitive performance between females and males among healthy controls (*n*/females = 336; *n*/males = 310), pediatric brain tumor (*n*/females = 72; *n*/males = 65) and hematopoietic and lymphoid tissues tumor survivors (*n*/females = 168; *n*/ males = 199).

Methods and Descriptive Statistics		Control				PBT				THL			
		Females	Males	*p*-Value	r	Females	Males	*p*-Value	r	Females	Males	*p*-Value	r
Digit Span Forward	Mean (SD)	8.20 (1.84)	8.07 (1.75)	0.324 (ns)	0.0388	7.76 (1.67)	8.14 (2.08)	0.406 (ns)	0.713	7.76 (1.78)	7.66 (1.96)	0.327 (ns)	0.0512
	Median	8	8			7	8			8	7		
Digit Span Backward	Mean (SD)	6.80 (1.85)	6.7 (1.79)	0.563 (ns)	0.0227	6.49 (1.78)	6.94 (2.44)	0.555 (ns)	0.0506	6.67 (1.89)	6.78 (1.87)	0.674 (ns)	0.0220
	Median	7	7			6	6			6	6		
COWAT	Mean (SD)	24.3 (10.1)	21.9 (8.44)	0.003 **	0.115	20.4 (10.9)	18.3 (9.82)	0.156 (ns)	0.121	20.8 (11.0)	21.9 (11.2)	0.387 (ns)	0.0452
	Median	24	22			19.5	16			19	20		
ROCF	Mean (SD)	27.0 (6.07)	25.3 (7.03)	0.005 **	0.112	20.7 (9.72)	20.6 (9.1)	0.855 (ns)	0.0158	22.5 (8.29)	24.2 (7.64)	0.059 (ns)	0.0988
	Median	28	27			22.5	22			23	24		
Grooved Pegboard	Mean (SD)	72.5 (19.1)	74.3 (18.2)	0.117 (ns)	0.0617	91.5 (31.8)	101 (33.5)	0.043 *	0.173	77.3 (20.8)	74.5 (20.4)	0.062 (ns)	0.0974
	Median	70	71			84	92			72	69		

Medium-to-large effect size (r ≥ 0.30). Control—healthy controls, PBT—pediatric brain tumor survivors, THL—hematopoietic and lymphoid tissues tumor survivors. COWAT—Controlled Oral Word Association Task, ROCF—the Rey–Osterrieth Complex Figure, F—females, M–males. *—Statistically significant difference, *p* < 0.005. **—Statistically significant difference, *p* < 0.001. ns—not significant.

## Data Availability

The data generated in this study are available upon request from the corresponding author.
